# Probiotic Potentiality from Versatile *Lactiplantibacillus plantarum* Strains as Resource to Enhance Freshwater Fish Health

**DOI:** 10.3390/microorganisms10020463

**Published:** 2022-02-17

**Authors:** Massimo Iorizzo, Gianluca Albanese, Francesco Letizia, Bruno Testa, Patrizio Tremonte, Franca Vergalito, Silvia Jane Lombardi, Mariantonietta Succi, Raffaele Coppola, Elena Sorrentino

**Affiliations:** Department of Agricultural, Environmental and Food Sciences, University of Molise, 86100 Campobasso, Italy; iorizzo@unimol.it (M.I.); g.albanese@studenti.unimol.it (G.A.); bruno.testa@unimol.it (B.T.); tremonte@unimol.it (P.T.); franca.vergalito@unimol.it (F.V.); silvia.lombardi@unimol.it (S.J.L.); succi@unimol.it (M.S.); coppola@unimol.it (R.C.)

**Keywords:** aquaculture, probiotic, *Lactiplantibacillus plantarum*, fish health

## Abstract

Dietary probiotic supplementation has the potential to enhance the health of fish and their disease resistance. In this study, some properties of ten *Lactiplantibacillus plantarum* strains have been evaluated, for their potential use as probiotics in freshwater fish diet. In particular, antimicrobial activity, antioxidant activity, the potentiality to survive the gastrointestinal transit and persist in the intestine, were evaluated in vitro. The experimental tests were carried out at 15 °C and 30 °C to determine the suitability of these lactic acid bacteria to be used as probiotics in the diet of fish grown at different temperatures. The results demonstrated that the evaluated *Lp. plantarum* strains, which often have significant differences among themselves, are characterized by important functional characteristics such as cell surface properties (auto-aggregation and hydrophobicity), ability to produce antioxidant substances, capacity to survive in the presence of 0.3% bile salts and acidic environment (2.5 pH), antagonistic activity against some fish opportunistic pathogens (*A. salmonicida*, *Ps. aeruginosa*, *E. coli* and *C. freundii*) and other unwanted bacteria present in fish products (*S. aureus* and *L. innocua*). The outcomes suggest that these *Lp. plantarum* strains may be candidates as probiotics in warm- and cold-water aquaculture.

## 1. Introduction

Aquaculture has become an important economic activity in many countries [1]. The current intensification of aquaculture has led to the promotion of conditions that favor the development of infection and disease-related problems [2]. Bacterial diseases in fish farming can cause high mortality, with subsequent economic losses [3]. The conventional approach so far applied in the mitigation, or cure, of bacterial diseases has been mainly based on the use of antibiotics [4].

The misuse of these compounds, however, is known to have several ancillary complications, such as negative effects on the gut microbiota [5,6] and antibiotic accumulation in edible products [7,8,9]. In addition, there is a general concern over the increased numbers of antibiotic-resistant bacteria in the environment [10,11].

As such, there is a growing concern to have other safe, non-antibiotic-based and eco-friendly alternatives for the treatment of the diseases [12]. In response to the reduction in antibiotic use in fish farming, vaccination has been playing a pivotal role in the control of infectious diseases in aquaculture for decades [13]. The wide acceptance of vaccines stems from the fact that there is no risk of drug resistance development in vaccinated animals and for the resulting protection of unvaccinated animals due to herd immunity. Conversely, the role of every vaccine is limited to the control and prevention of a single infectious disease [14]. Subsequently, a single approach is not sufficient to maintain the health status of fish in aquaculture, but rather a combination of different strategies is required.

In recent years, the use of probiotics as agents of biological control is a promising alternative approach for the control of infectious agents and treatment of diseases [15,16]. Although probiotics were initially used for disease control, their use in aquaculture has now extended for further reasons: improvement of fish growth and reproduction, enzymatic contribution to digestion, modulation of the gut microbiota, enhancement of hematological parameters and immune response [15,16,17,18,19]. Lactic acid bacteria (LAB) have gained considerable attention as probiotics in aquaculture, and over the last two decades, numerous investigations were carried out to evaluate the probiotic properties of different genera and species [20,21,22,23]. Based on these studies, it is evident that LABs along with other bacteria that belong to the indigenous microbiota play an important role in improving the health and welfare of fish. Among several probiotic bacterial species, several reports have been published on the beneficial role of *Lactiplantibacillus plantarum* (previously *Lactobacillus plantarum*) and its use as a probiotic in aquaculture [24,25,26,27]. *Lp. plantarum* is a bacterium capable of colonizing several ecological niches, including the intestinal tract of mammals, insects, and fishes [28,29,30]. Many studies have shown that some *Lp. plantarum* strains that inhabit our intestinal tract must have intrinsic resistance mechanisms to resist the gastrointestinal transit [31,32]. Recently, several studies have suggested that *Lp. plantarum* is a versatile LAB, among the most important due to its distinctive probiotic properties, as it can tolerate acid and bile conditions as well as exhibit antioxidant and antimicrobial activities [33,34,35,36,37,38,39]. Therefore, some strains of *Lp. plantarum* are proposed or used as probiotics in aquaculture practices [40,41,42]. Considering the positive impact of probiotics, it is important to select new specific strains to be used in freshwater fish aquaculture [20,43].

This study focused on some functional and probiotic activities of ten *Lp. plantarum* strains, to assess their suitability to be used as probiotics in aquaculture. In particular, antioxidant and antimicrobial activities, cell surface properties (auto-aggregation and hydrophobicity) and capability to survive at low pH and in the presence of bile salts were studied. Our tests were conducted at temperatures of 15 °C and 30 °C degrees.

The reason for this experimental choice was to evaluate the versatility of the selected *Lp. plantarum* strains and their possible use as probiotics in the diet of farmed fish grown at different temperatures.

## 2. Materials and Methods

### 2.1. LAB Strains

Ten *Lp. plantarum* strains (23V, 33V, 36V, 37V, 64V, 65V, 66V, 67V, 68V, 73V) isolated from the intestinal tract of the Mediterranean trout (*Salmo macrostigma*) were used [30]. Their 16S rRNA sequences have been deposited in GenBank under accession numbers from MZ452092 to MZ452095 and from MZ452098 to MZ452103.

### 2.2. Antimicrobial Activity

The antimicrobial activity of the *Lp. plantarum* strains (producers) was evaluated against the following indicator bacteria: *Escherichia coli* ATCC 11775, *Listeria innocua* ATCC 33090, *Proteus mirabilis* ATCC 29906, *Staphylococcus aureus* ATCC 29213, *Pseudomonas aeruginosa* ATCC 27853, *Citrobacter freundii* ATCC 8090, and *Aeromonas salmonicida* ATCC 33658 belonging to the American Type Culture Collection (ATCC Manassas, VA, USA).

The *Lp. plantarum* strains were grown in MRS broth (Oxoid Ltd., Hampshire, UK). After 16 h at 30 °C, the broth culture of every single strain was centrifugated (8000× *g* rpm for 10 min at 4 °C) to separate the cell pellet from the cell-free supernatant (CFS). Before being used, the CFS was mechanically sterilized by means of filtration (cellulose acetate membrane, pore size 0.22 μm (Sigma-Aldrich; St. Louis, M0, USA).

The antimicrobial activity of CFSs were tested in accordance with the Kaewchomphunuch et al. protocol [44]. As a negative control, 50 μL of MRS, adjusted to pH 3.8 with hydrochloric acid 1N (Sigma-Aldrich), was used. Antibacterial activity has been evaluated after 72 h, at 15 °C and 30 °C, measuring the diameter (mm) of the clear zone of inhibition (ZOI) around the inoculated wells [44]. The tests were conducted in triplicate.

### 2.3. Evaluation of Acid and Bile Tolerance

The *Lp. plantarum* strains were anaerobically grown in MRS for 18 h at 30 °C. The cells were collected by centrifugation, washed twice with physiological solution (NaCl 0.9%), and suspended in MRS broth. In order to assess the ability of bacteria to survive acidic environments, 100 µL of bacterial suspension were transferred in test tubes containing 10 mL of MRS adjusted to pH 2.5 with 1 M HCl (Sigma-Aldrich; St. Louis, MO, USA). Similarly, to determine the bile tolerance, 100 µL of bacterial suspension were transferred in test tubes containing 10 mL of MRS enriched with 0.3% (*w*/*v*) of bile salts (cholic acid sodium salt, 50%; deoxycholic acid sodium salt, 50% Sigma-Aldrich) [44].

Finally, the test tubes were incubated at 15 °C and 30 °C in anaerobiosis. Viable cell counts were performed at the beginning of the trial and after 3 and 24 h by plate counting on MRS agar incubated anaerobically at 30 °C for 48 h. The experiments were performed in triplicate and expressed as mean values.

### 2.4. Cell Surface Properties

#### 2.4.1. Bacterial Cultures

The bacterial strains were grown in MRS broth at 30 °C. After 12 h, the cultures were centrifugated (8000× *g* rpm for 15 min at 4 °C), washed twice and resuspended in a sterile phosphate buffer saline (PBS, pH 7) to an optical density of 0.5 MacFarland scale (OD_580_) to standardize the bacterial density at 10^8^ CFU/mL [45]. The OD_580_ of bacterial suspension (BS) was measured using a spectrophotometer (Multilabel Counter–PerkinElmer 1420, San Jose, CA, USA). The tests were then conducted in triplicate.

#### 2.4.2. Auto-Aggregation

The auto-aggregation (AA) assay was performed according to Iorizzo et al. [31]. The absorbance measurements (OD_580_) of the BSs were carried out after 1, 2, 5, and 24 h at 15 °C and 30 °C. The AA (%) was obtained using the following formula: AA% = (1 − OD_t_/OD_0_) × 100 [46], where OD_0_ is the initial absorbance and OD_t_ is the absorbance detected after 1, 2, 5, and 24 h.

#### 2.4.3. Hydrophobicity

The percentage hydrophobicity has been determined considering the capacity of *Lp. plantarum* strains to adhere to hydrocarbons (BATH) using xylene and toluene [47]. The BS was added with 50% of every single hydrocarbon and the two-phase system was vortexed for 5 min. After 15, 30, and 60 min of stationary phase at 15 °C and 30 °C, the aqueous phase was carefully removed and spectrophotometric readings were carried out using the wavelength control set to 580 nm. Hydrophobicity (H) was calculated as a percentage decrease in optical density and was expressed using the following formula: H% = (1 − OD_t_/OD_0_) × 100) [46], where OD_t_ represents the absorbance after 15, 30, and 60 min, while OD_0_ represents the optical density value before adding the hydrocarbon.

### 2.5. Antioxidant Activity

The overnight cultures (10^7^ CFU/mL) of the *Lp. plantarum* strains in 10 mL of MRS medium were centrifuged at 8000× *g* rpm for 5 min at 4 °C.

Cell pellets (CP) were fractionated into two aliquots for the protein contents and antioxidant activity. Total cell protein extraction was carried out resuspending the CP in 1 mL of NaOH 0.1 M, treated with heat at 95 °C for 20 min and stored at 4 °C. After 24 h, samples were centrifugated at 13,000× *g* rpm for 15 min at 4 °C and the supernatants were used for protein quantification according to Di Martino et al. [48]. The cell protein was expressed as mg/mL, using bovine serum albumin (BSA) as the standard.

For the antioxidant activity, the cell pellet was washed twice with sterile water and resuspended in 500 µL of cold pure methanol, and after 12 h of storage at −20 °C, centrifuged at 13,000× *g* rpm for 15 min at 4 °C. The supernatants of cell extract (CES) were used for the evaluation of the antioxidant activity.

Antioxidant activity of the CES was evaluated using the 2,2 azino-bis 3-ethylbenzothiazoline-6-sulfonic acid (ABTS·+) radical cation method according to Re et al. [49] and scavenging of 2,2-diphenyl-1-picrylhydrazyl (DPPH) free radical according to the method described by Aarti et al. [50], with some modifications. ABTS radical cations (ABTS·+) were produced by reacting the ABTS methanol solution 7 mM with 2.45 mM potassium persulfate, storing it in the dark at room temperature for 24 h before use. The ABTS·+ solution was diluted with cold pure methanol to an optical density (OD) of 0.700 at 745 nm. Then, 100 µL of CES were mixed with 900 µL of the ABTS·+ solution. The OD was measured at 745 nm after 6 min in the dark at room temperature using a BioSpectrometer (Eppendorf).

For the DPPH radical-scavenging capacity, 100 µL of CES were mixed with 900 µL of DPPH radical solution (0.05 mM) and diluted with cold pure methanol to OD of 0.700 at 515 nm after 15 min.

For ABTS and DPPH assays, Trolox was used as the standard for the calibration curve.

The antioxidant activity was expressed as the ratio (*w*/*w*) between μg/mL Trolox and mg/mL of total cell protein (BSA equivalents). All the reagents used for this experiment were from Sigma-Aldrich.

### 2.6. Statistical Analysis

All the data obtained from the three independent experiments are expressed as mean ± standard deviation (SD). Statistical analysis was performed using an analysis of variance (ANOVA) followed by Tukey’s multiple comparison test. Statistical significance was attributed to *p*-values < 0.05 using SPSS software (IBM SPSS Statistics 21) for the analysis.

## 3. Results

### 3.1. Antimicrobial Activity

The results of the antagonistic activity of the *Lp*. *plantarum* strains against indicator bacteria at 15 °C and 30 °C are presented in Table 1 and Table 2 as the mean diameter (mm) of the growth inhibition zone (ZOI). Tests using the acidified MRS pH 3.8 showed no inhibitory effect. At 30 °C, all the CFSs of the *Lp. plantarum* strains inhibited the growth of indicator bacteria causing ZOI between 5 and 14 mm, in some cases with statistically significant differences. At 15 °C, the overall results show there was a greater diversification of antagonistic activity than that caused at 30 °C. The results of the antimicrobial activity conducted at 30 °C showed the ability of the ten *Lp. plantarum* to inhibit all indicator strains. The specific data at 15 °C showed an inhibitory effect of all tested *Lp. plantarum* strains against *A. salmonicida*, *Ps. aeruginosa, E. coli*, and *C. freundii,* while only *Lp. plantarum* 66V, 67V, 68V, and 73V were able to partially inhibit the development of *S. aureus* and *L. innocua*.

### 3.2. pH and Bile Resistance

To survive and persist in the gastrointestinal tract, probiotic candidates must be able to survive in an acidic environment and in the presence of bile salts. The results of the effect of acid and bile stress on the survival of the *Lp. plantarum* strains are reported in Tables S1 and S2 (Supplementary Materials). All the examined strains exhibited high resistance in an acidic environment (pH 2.5) and the presence of bile salts (0.3%) with significant differences between them.

The viability of lactobacilli, in MRS at pH 2.5 after 2 h of incubation, from the initial number of 8.00 log CFU/mL decreases approximately only 1 log cycle at 15 °C and 30 °C. The general comparison of all strains examined, for significant differences in the cell viability, indicated similar pH tolerance. The bile salts present in bacterial cultures were much less effective on bacterial viability than the effect of pH 2.5. The selected *Lp. plantarum* strains were able to survive in the presence of 0.3% bile salts. In fact, over 24 h, they maintained almost entirely the initial viability (8 log CFU/mL) both at 15 °C and at 30 °C.

### 3.3. Cell Surface Properties: Hydrophobicity and Auto-Aggregation

**Figure 1:**
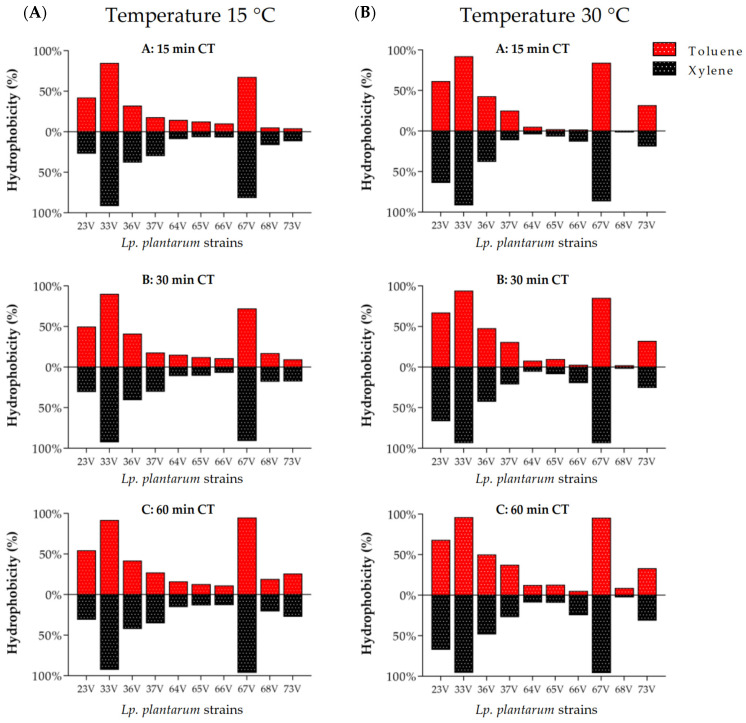
Adhesion of the *Lp. plantarum* strains to toluene and xylene expressed as hydrophobicity (%) after 15, 30, and 60 min contact time (CT) at 15 °C (**A**) and 30 °C (**B**).

The hydrophobicity was investigated using the ability of the bacteria to adhere at 15 °C and 30 °C to toluene and xylene hydrocarbons. The hydrophobicity (%) of the *Lp. plantarum* strains is reported graphically in Figure 1 and numerically in Table S3 (Supplementary Materials). In all tests, we have detected significant differences among the tested *Lp. plantarum* strains. For all strains, the adhesion to hydrocarbons increased gradually during the test period (60 min). *Lp. plantarum* 33V and 67V strains, already after 15 min both at 15 °C and 30 °C, showed a high adherence to toluene and xylene with a hydrophobicity percentage greater than 66%, and after 60 min, the percentage was greater than 90%. After 60 min at 15 °C, *Lp. plantarum* 23V and 36V adhered to toluene with a hydrophobicity percentage of 41.4% and 54%, respectively, while for the other strains we found a hydrophobicity of less than 30%. At 30 °C, *Lp. plantarum* 23V and 36V adhered to toluene with percentages of 49.5% and 67.5%, respectively; the other strains did not show a percentage of hydrophobicity greater than 37%. In the test with xylene after 60 min, except for *Lp. plantarum* 33V and 67V, the other strains did not exceed the hydrophobicity percentages obtained from *Lp. plantarum* 36V: 49.5% at 30 °C and 48.1% at 15 °C.

**Figure 2:**
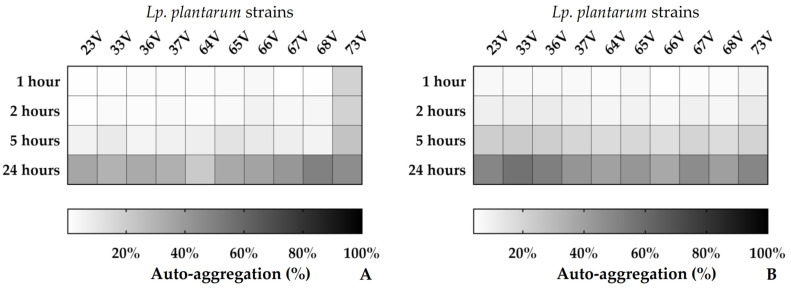
Auto-aggregation (AA%) of the *Lp. plantarum* strains at 15 °C (**A**) and 30 °C (**B**).

The AA results are depicted in Figure 2, and the relative numeric data are presented in Table S4 (Supplementary Materials). The data highlighted significant differences among the tested *Lp. plantarum* strains. The tests showed that the ability to aggregate and sediment increased progressively, until reaching, after 24 h, range values between 20% and 50% at 15 °C and between 36% and 57% at 30 °C.

### 3.4. Antioxidant Activity

The antioxidant activities, expressed as the ratio between μg Trolox/mL and mg/mL of cell protein (BSA equivalents), are shown in Table 3.

The values obtained in ABTS assays were between 22.6 (37V strain) and 38.0 (65V strain), while the values obtained in DPPH assays were between 1.0 (23V strain) and 2.2 (67V strain).

## 4. Discussion

Fish’s digestive tract is colonized by a wide microbial community that has important influences on the immune system, nutrient assimilation, and a wide range of other host activities. The gut microbiota can positively or negatively interact with each other. The interactions established among the components of the microbiota can significantly affect fish health. Fish become more susceptible to pathogens due to their stressful environment, which eventually suppresses their immune system, causing dysbiosis, which can lead to opportunistic infections that would be otherwise suppressed. Among all pathogens, bacteria are the most prominent disease-causing agents in fish and cause major problems and economic losses in commercial farming of a large number of cultivated fish species [51].

Conversely, an aggressive pathogen producing powerful virulence factors may be able to disrupt the normal microbial balance and lead to dysbiosis. Most surveys on probiotic applications in aquaculture consider the ability to inhibit pathogenic bacteria [52].

In this field, we evaluated the antagonistic activity of ten *Lp. plantarum* strains against some opportunistic fish pathogens (*C. freundii*, *A. salmonicida*, *P. mirabilis*, *Ps. aeruginosa*) and other unwanted bacteria present in fish products [6,53,54,55,56]. It is well known that some LAB strains may protect fish from intestinal pathogens by several possible mechanisms, including the production of inhibitory substances, such as organic acids, hydrogen peroxide, bacteriocins, and carbon peroxide [20,57].

Our results on the antimicrobial activity indicated that at 15 and 30 °C, the MRS pH 3.8 did not inhibit the growth of indicator bacteria. The data obtained in our study seem to exclude that antibacterial activity is due to the acidic environment. Therefore, the antagonistic activity highlighted in our tests is probably due to substances produced by lactobacilli during the growth phase in the native CFS [58]. The nature of these substances needs to be investigated in the future. The ability to inhibit pathogens is a desirable property for probiotics and a sustainable alternative to antibiotics; dietary supplementation with these LABs may provide effective prophylaxis against infections in fish [59,60]. The fish, like other living organisms, developed an enzymatic antioxidant system including different enzymes (e.g., superoxide dismutase, glutathione peroxidase, glutathione reductase, and catalase) and a non-enzyme antioxidant system based on the production of different compounds as glutathione, thioredoxin, vitamin C, and vitamin E [61,62]. This complex system is capable of providing the balance between production and removal of reactive oxygen species (ROS) under normal physiological conditions. In fish, like other organisms, the lack of balance between the production of ROS and the antioxidant defense system can cause the oxidation of biological macromolecules, inducing cell damage [63]. Excessive production of ROS in fish can be caused by changes in environmental O_2_ (hyperoxia and hypoxia), temperature (hyper or hypothermia), and malnutrition [64]. In addition, the presence in the aquatic environment of heavy metals, pesticides (insecticides, herbicides, and fungicides), along with oil products, induces oxidative stress on fish [65]. In fact, various research studies proposed the use of new and safe natural antioxidants as an alternative for synthetic antioxidants [66]. Other studies in aquaculture have shown promising antioxidative effects of beneficial additives, such as probiotics, on different fish species [67,68]. Recently, LAB have been considered for their antioxidant activity [66,68,69]. Based on this evidence, in our research, we examined the antioxidant activity of selected *Lp. plantarum* strains using the ABTS and DPPH methods. These two techniques may be utilized in aqueous and non-polar organic solvents to examine both hydrophilic and lipophilic antioxidants [70,71,72,73]. The obtained results showed that the selected *Lp. plantarum* strains possess antioxidant activity, confirming what has been reported in other studies conducted on this species [74,75,76,77].

The DPPH method provided lower values than the ABTS test. This difference is probably because it has a different sensitivity to the different antioxidant substances present in the CES, which needs to be investigated in further detail.

Consumption of probiotics alone, or foods supplemented with probiotics, may reduce oxidative damage in fish cells. The health-benefiting properties of probiotics are largely dependent on their prolonged residence in the GIT and are dictated by adherence to the intestinal mucosa. The ability of probiotic bacteria to adhere to intestinal epithelial cells involves various surface properties, including hydrophobicity and auto-aggregation [15,16,78]. These characteristics promote the colonization and permanence of probiotics in the gastrointestinal tract (GIT). Moreover, the examined *Lp. plantarum* strains had values of auto-aggregation and hydrophobicity in line with previous studies [47,79]. In particular, *Lp. plantarum* 33V and 67V strains stood out for their performances. Our data confirm that some *Lp. plantarum* strains have the potential to survive in the gastrointestinal tract and adhere to its epithelial cells [80,81]. An effective probiotic should be viable, safe, bile- and gastric-juices-tolerant, able to survive through the gastrointestinal tract, adhere and colonize gut epithelial cells. Our study showed a high survival of the 10 strains of *Lp. plantarum* in an acidic environment (pH 2.5) and the presence of 0.3% of bile salts. These results showed that some *Lp. plantarum* strains had a high likelihood to survive stress in the gastrointestinal tract like low pH and bile presence, as highlighted in a previous study by Bucio et al. [82]. Gut transit time in fish depends on numerous factors including species, fish age/size, water temperature, food quality, meal size and feeding frequency [83,84]; water temperature influences gut transit time and nutrient digestibility as it can correlate to feed intake and enzyme activity [85,86]. This evidence motivated us to conduct bile tolerance tests for 24 h. In addition, our tests were conducted at temperatures of 15 °C and 30 °C degrees. Fish are poikilotherms, they do not have a thermoregulatory system and their body temperature fluctuates in response to the temperature of the surrounding environment. Certain fish species are classified as cold-water fish, while others as warm-water or tropical fish; while cold-water fish are rarely exposed to temperatures greater than 20 °C, tropical fish are frequently exposed to temperatures well above 30 °C [87].

Our results showed that the selected *Lp. plantarum* strains have high adaptability to temperature, confirming the versatility of this species to adapt to different environmental conditions [88]. This characteristic would make it possible to use them in the diet of different fish species as well as make them tolerant to the different annual climatic seasons. The seasonal changes in water temperature can be extremely different for cold-water and warm-water species, they can range from below 5 °C to 19 °C and 16 °C to 39 °C, respectively [89,90].

## 5. Conclusions

The supplementation of fish diet with specific probiotic microorganisms can contribute to improving the welfare and disease resistance, which is a widespread problem in aquaculture. The present study improves the application knowledge for the implementation of *Lp. plantarum* as probiotic in freshwater fish diet. Our results demonstrated that the examined *Lp. plantarum* strains are characterized by important features such as antioxidant activity, ability to survive in the presence of bile, tolerance to low pH, cell surface properties and antagonism activity against some fish pathogens. Therefore, these LABs meet some important criteria to be candidates as probiotics.

## Figures and Tables

**Table 1:** Antimicrobial activity at 15 °C by cell-free supernatant (CFS) of the tested *Lp. plantarum* strains against different indicator bacteria. The data (mean ± SD; *n* = 3) are expressed as zone of inhibition-ZOI (mm). Different lowercase letters (a–d) in each row indicate significant differences (*p* < 0.05).

Indicator Strains	*Lp. plantarum* Strains									
	23V	33V	36V	37V	64V	65V	66V	67V	68V	73V
*L. innocua*	0 ± 0 ^d^	0 ± 0 ^d^	0 ± 0 ^d^	0 ± 0 ^d^	0 ± 0 ^d^	0 ± 0 ^d^	7.1 ± 0.2 ^a^	5.0 ± 0.3 ^c^	5.5 ± 0.4 ^b^	5.9 ± 0.5 ^b^
*A. salmonicida*	7.0 ± 0.6 ^b^	7.0 ± 0.5 ^b^	7.7 ± 0.6 ^a^	7.5 ± 0.6 ^a^	8.5 ± 0.3 ^a^	7.3 ± 0.3 ^a^	6.6 ± 0.6 ^b^	7.5 ± 0.3 ^a^	8.0 ± 0.2 ^a^	7.7 ± 0.6 ^a^
*C. freundii*	7.2 ± 0.7 ^c^	7.2 ± 0.6 ^c^	8.0 ± 0.4 ^b^	6.4 ± 0.6 ^c^	9.9 ± 0.4 ^a^	7.0 ± 0.4 ^c^	10.5 ± 0.4 ^a^	9.0 ± 0.6 ^b^	10.2 ± 0.3 ^a^	10.3 ± 0.5 ^a^
*P. mirabilis*	9.2 ± 0.6 ^a^	7.4 ± 0.6 ^b^	8.3 ± 0.5 ^b^	9.7 ± 0.4 ^a^	9.5 ± 0.5 ^a^	9.0 ± 0.2 ^a^	9.6 ± 0.4 ^a^	10.1 ± 0.2 ^a^	9.5 ± 0.4 ^a^	7.3 ± 0.6 ^b^
*S. aureus*	0 ± 0 ^c^	0 ± 0 ^c^	0 ± 0 ^c^	0 ± 0 ^c^	0 ± 0 ^c^	0 ± 0 ^c^	6.5 ± 0.4 ^b^	7.1 ± 0.5 ^b^	8.9 ± 0.3 ^a^	6.9 ± 0.4 ^b^
*Ps. aeruginosa*	7.0 ± 0.3 ^a^	8.0 ± 0.3 ^a^	7.9 ± 0.6 ^a^	8.0 ± 0.7 ^a^	7.9 ± 0.6 ^a^	6.8 ± 0.3 ^a^	6.0 ± 0.1 ^b^	7.3 ± 0.4 ^a^	7.8 ± 0.4 ^a^	5.8 ± 0.2 ^b^
*E. coli*	8.8 ± 0.6 ^b^	9.9 ± 0.6 ^a^	9.6 ± 0.5 ^a^	10.1 ± 0.1 ^a^	9.8 ± 0.2 ^a^	6.9 ± 0.4 ^c^	7.0 ± 0.3 ^c^	7.8 ± 0.3 ^b^	8.9 ± 0.6 ^a^	7.1 ± 0.1 ^c^

**Table 2:** Antimicrobial activity at 30 °C by cell-free supernatant (CFS) of the tested *Lp. plantarum* strains against different indicator bacteria. The data (mean ± SD; *n* = 3) are expressed as zone of inhibition-ZOI (mm). Different lowercase letters (a–c) in each row indicate significant differences (*p* < 0.05).

Indicator Strains	*Lp. plantarum* Strains									
	23V	33V	36V	37V	64V	65V	66V	67V	68V	73V
*L. innocua*	7.9 ± 0.3 ^b^	7.9 ± 0.2 ^b^	7.0 ± 0.4 ^c^	8.0 ± 0.3 ^b^	9.0 ± 0.3 ^a^	9.0 ± 0.3 ^a^	5.9 ± 0.4 ^c^	10.0 ± 0.5 ^a^	8.2 ± 0.8 ^b^	9.0 ± 0.2 ^a^
*A. salmonicida*	7.8 ±0.6 ^a^	8.2 ± 0.6 ^a^	8.5 ± 0.5 ^a^	8.9 ± 0.3 ^a^	8.4 ± 0.5 ^a^	7.2 ± 0.8 ^b^	7.4 ± 1.0 ^a^	7.7 ± 0.5 ^a^	7.2 ± 0.4 ^b^	7.9 ± 0.2 ^a^
*C. freundii*	7.9 ± 0.7 ^b^	8.7 ± 0.8 ^b^	8.8 ± 0.9 ^b^	9.8 ± 1.2 ^b^	14.0 ± 0.8 ^a^	9.1 ± 0.8 ^b^	7.9 ± 0.3 ^b^	7.9 ± 0.4 ^b^	8.9 ± 0.6 ^b^	6.7 ± 0.8 ^c^
*P. mirabilis*	7.0 ± 0.5 ^a^	8.0 ± 0.3 ^a^	8.0 ± 0.4 ^a^	7.1 ± 0.6 ^a^	6.1 ± 0.8 ^b^	6.9 ± 0.6 ^a^	5.2 ± 0.5 ^b^	5.8 ± 0.4 ^b^	6.4 ± 0.5 ^b^	7.0 ± 0.6 ^a^
*S. aureus*	5.9 ± 0.7 ^b^	5.9 ± 0.6 ^b^	7.0 ± 0.4 ^a^	6.8 ± 0.2 ^a^	6.8 ± 0.6 ^a^	6.7 ± 0.2 ^a^	7.0 ± 0.6 ^a^	7.9 ± 0.6 ^a^	7.2 ± 0.4 ^a^	6.2 ± 0.6 ^b^
*Ps. aeruginosa*	7.2 ± 0.9 ^a^	7.0 ± 0.9 ^a^	6.4 ± 0.5 ^a^	6.8 ± 0.6 ^a^	6.9 ± 0.5 ^a^	6.0 ± 0.6 ^a^	5.8 ± 0.5 ^a^	7.4 ± 0.4 ^a^	7.0 ± 0.3 ^a^	6.0 ± 0.5 ^a^
*E. coli*	9.0 ± 0.5 ^a^	9.0 ± 0.6 ^a^	8.6 ± 0.4 ^a^	9.9 ± 1.0 ^a^	9.9 ± 1.0 ^a^	10.2 ± 1.0 ^a^	8.0 ± 0.8 ^b^	7.9 ± 0.5 ^b^	8.0 ± 0.7 ^b^	7.0 ± 0.6 ^b^

**Table 3:** Antioxidant activity of the *Lp. plantarum* strains. All values are expressed as mean ± standard deviation (*n* = 3). Different lowercase letters (a–d) in each row indicate significant differences (*p* < 0.05).

Antioxidant Assay	*Lp. plantarum* Strains									
	23V	33V	36V	37V	64V	65V	66V	67V	68V	73V
ABTS	24.1 ± 0.3 ^d^	30.9 ± 0.7 ^b^	31.5 ± 1.1 ^b^	22.6 ± 0.2 ^d^	31.0 ± 1.1 ^b^	38.0 ± 0.7 ^a^	29.1 ± 0.9 ^b^	31.2 ± 1.4 ^b^	27.4 ± 2.3 ^c^	25.9 ± 0.6 ^c^
DPPH	1.0 ± 0.7 ^b^	1.3 ± 0.3 ^a^	1.6 ± 0.1 ^a^	0.8 ± 0.4 ^b^	1.3 ± 0.1 ^a^	1.5 ± 0.6 ^a^	1.0 ± 0.4 ^b^	2.2 ± 0.1 ^a^	2.1 ± 0.3 ^a^	1.2 ± 0.1 ^a^

μg Trolox/mg cell proteins (BSA eq.).

## Data Availability

The data presented in this study are available in the Supplementary Materials.

## Supplementary Materials

The following supporting information can be downloaded at: https://www.mdpi.com/article/10.3390/microorganisms10020463/s1, Table S1: Resistance to pH 2.5 at 15 °C and 30 °C, Table S2: Resistance to 0.3% bile salts at 15 °C and 30 °C, Table S3: Adhesion to toluene and xylene of *Lp. plantarum* strains after different contact times; Table S4: Auto-aggregation (AA) at 15 °C and 30 °C of the *Lp. plantarum* strains.
