# Supplementary material for: Probiotic Potentiality from Versatile Lactiplantibacillus plantarum Strains as Resource to Enhance Freshwater Fish Health
Source: Microorganisms. 2022 Feb 17;10(2):463. doi: 10.3390/microorganisms10020463 (PMC8877946; doi:10.3390/microorganisms10020463)
Supplement: Supplementary file 1 [file microorganisms-10-00463-s001.zip › Supplementary material/Caption Supplementary material.pdf]

Table S1 Resistance to pH 2.5 at 15°C and 30°C of the examined *Lp. plantarum* strains (23V, 33V, 36V, 37V, 64V, 65V, 66V, 67V, 68V, 73V). The data (mean ± SD; n=3) were expressed as log CFU/mL. Different lowercase letters (a–h) in each row indicate significant differences ($p < 0.05$).

Table S2 Resistance to 0.3% bile salts at 15°C and 30°C of the examined *Lp. plantarum* strains (23V, 33V, 36V, 37V, 64V, 65V, 66V, 67V, 68V, 73V). The data (mean ± SD; n=3) were expressed as log CFU/mL. Different lowercase letters (a–e) in each row indicate significant differences ($p < 0.05$).

Table S3 Adhesion to toluene and xylene of *Lp. plantarum* strains (23V, 33V, 36V, 37V, 64V, 65V, 66V, 67V, 68V, 73V) after different contact times. The data (mean ± SD; n=3) were expressed as percentage of hydrophobicity

Table S4 Auto-aggregation (AA) at 15°C and 30°C of the *Lp. plantarum* strains (23V, 33V, 36V, 37V, 64V, 65V, 66V, 67V, 68V, 73V). The data (mean ± SD; n=3) were expressed as percentage of AA
