# Supplementary material for: Probiotic Potentiality from Versatile Lactiplantibacillus plantarum Strains as Resource to Enhance Freshwater Fish Health
Source: Microorganisms. 2022 Feb 17;10(2):463. doi: 10.3390/microorganisms10020463 (PMC8877946; doi:10.3390/microorganisms10020463)
Supplement: Supplementary file 1 [file microorganisms-10-00463-s001.zip › Supplementary material/S1 pH resistance.pdf]

Table S1 Resistance to pH 2.5 at 15°C and 30°C of the examined *Lp. plantarum* strains (23V, 33V, 36V, 37V, 64V, 65V, 66V, 67V, 68V, 73V). The data (mean ± SD; n=3) were expressed as log CFU/mL. Different lowercase letters (a–h) in each row indicate significant differences ($p < 0.05$).

Temperature (°C)	Time (h)	*Lp. Plantarum* strain									
		23V	33V	36V	37V	64V	65V	66V	67V	68V	73V
15	0	8.43 ± 0.01^{c}	8.15 ± 0.01^{e}	8.12 ± 0.01^{f}	8.27 ± 0.06^{d}	8.22 ± 0.03^{e}	8.33 ± 0.04^{d}	8.71 ± 0.02^{a}	8.34 ± 0.05^{c}	8.52 ± 0.03^{b}	8.60 ± 0.04^{b}
	3	7.15 ± 0.03^{f}	7.38 ± 0.01^{d}	7.41 ± 0.01^{c}	6.87 ± 0.05^{h}	7.21 ± 0.04^{e}	7.49 ± 0.02^{c}	7.25 ± 0.01^{e}	7.05 ± 0.05^{g}	7.64 ± 0.04^{b}	7.78 ± 0.04^{a}
30	0	8.32 ± 0.04^{a}	8.16 ± 0.02^{b}	8.22 ± 0.06^{b}	8.22 ± 0.04^{b}	8.22 ± 0.02^{b}	8.29 ± 0.02^{a}	8.26 ± 0.05^{b}	8.34 ± 0.03^{a}	8.40 ± 0.02^{a}	8.39 ± 0.03^{a}
	3	7.66 ± 0.03^{b}	7.83 ± 0.02^{a}	7.50 ± 0.07^{c}	7.24 ± 0.03^{d}	7.39 ± 0.03^{c}	7.61 ± 0.04^{b}	7.57 ± 0.04^{b}	7.32 ± 0.03^{d}	7.60 ± 0.01^{b}	7.89 ± 0.06^{a}
