# Supplementary material for: Probiotic Potentiality from Versatile Lactiplantibacillus plantarum Strains as Resource to Enhance Freshwater Fish Health
Source: Microorganisms. 2022 Feb 17;10(2):463. doi: 10.3390/microorganisms10020463 (PMC8877946; doi:10.3390/microorganisms10020463)
Supplement: Supplementary file 1 [file microorganisms-10-00463-s001.zip › Supplementary material/S2 bile salt resistance.pdf]

Table S2 Resistance to 0.3% bile salts at 15°C and 30°C of the examined *Lp. plantarum* strains (23V, 33V, 36V, 37V, 64V, 65V, 66V, 67V, 68V, 73V). The data (mean ± SD; n=3) were expressed as log CFU/mL. Different lowercase letters (a–e) in each row indicate significant differences ($p < 0.05$).

Temperature (°C)	Time (h)	***Lp. plantarum*** **strains**									
		**23V**	**33V**	**36V**	**37V**	**64V**	**65V**	**66V**	**67V**	**68V**	**73V**
15	3	8.83±0.04^a	8.85±0.05^a	8.72±0.05^b	8.75±0.04^b	8.71±0.01^b	8.27±0.02^c	8.62±0.01^c	8.80±0.03^a	8.33±0.02^d	8.31±0.03^c
	24	8.71±0.05^a	8.62±0.03^a	8.55±0.04^b	8.11±0.05^d	8.20±0.01^d	8.25±0.04^c	8.33±0.02^c	8.58±0.03^b	8.12±0.04^d	8.08±0.07^e
30	3	8.84±0.06^a	8.11±0.03^c	8.79±0.01^a	8.79±0.03^a	8.17±0.02^c	8.15±0.01^c	8.14±0.03^c	8.68±0.01^b	8.14±0.04^c	8.73±0.02^b
	24	8.34±0.06^b	8.36±0.02^b	8.78±0.01^a	8.32±0.02^b	8.35±0.04^b	8.34±0.04^b	8.10±0.04^c	8.06±0.03^c	8.07±0.03^c	8.36±0.01^b
