# Supplementary material for: Probiotic Potentiality from Versatile Lactiplantibacillus plantarum Strains as Resource to Enhance Freshwater Fish Health
Source: Microorganisms. 2022 Feb 17;10(2):463. doi: 10.3390/microorganisms10020463 (PMC8877946; doi:10.3390/microorganisms10020463)
Supplement: Supplementary file 1 [file microorganisms-10-00463-s001.zip › Supplementary material/S3 hydrophobicity.pdf]

Table S3 Adhesion to toluene and xylene of *Lp. plantarum* strains (23V, 33V, 36V, 37V, 64V, 65V, 66V, 67V, 68V, 73V) after different contact times. The data (mean ± SD; n=3) were expressed as percentage of hydrophobicity

**Hydrophobicity (%) Toluene 15 °C**										
**Time (min)**	**23V**	**33V**	**36V**	**37V**	**64V**	**65V**	**66V**	**67V**	**68V**	**73V**
15	41.5±2.3	84.3±3.5	31.5±2.4	17.3±1.7	14.1±2.2	11.9±1.8	9.73±0.8	66.9±3.0	4.8±1.0	3.5±0.5
30	49.2±1.8	89.6±1.4	40.8±2.8	17.3±1.1	14.5±1.4	11.7±1.9	10.2±0.6	71.8±3.7	16.5±1.4	9.0±0.4
60	54.0±1.9	91.4±1.3	41.4±1.5	26.8±1.9	15.8±1.0	12.4±0.4	10.5±0.8	94.4±2.3	18.5±0.8	25.4±2.8

**Hydrophobicity (%) Xylene 15 °C**										
**Time (min)**	**23V**	**33V**	**36V**	**37V**	**64V**	**65V**	**66V**	**67V**	**68V**	**73V**
15	26.8±2.0	91.3±1.3	37.7±2.4	29.6±2.0	8.6±0.6	6.23±0.7	6.6±0.6	81.4±3.7	16.1±2.3	11.5±0.6
30	30.3±3.0	92.3±2.3	40.5±2.9	29.9±0.8	10.8±0.6	10.5±1.4	6.6±0.4	90.6±2.6	17.8±0.7	17.5±3.5
60	30.8±3.2	92.4±4.0	42.0±1.4	34.9±3.8	15.1±2.0	13.1±1.0	12.7±2.2	96.1±1.5	20.3±1.2	27.1±2.0

**Hydrophobicity (%) Toluene 30 °C**										
**Time (min)**	**23V**	**33V**	**36V**	**37V**	**64V**	**65V**	**66V**	**67V**	**68V**	**73V**
15	61.1±2.2	91.8±3.3	42.3±1.8	24.7±1.7	4.6±0.4	1.6±0.3	1.4±0.4	83.8±3.0	0±0	31.3±4.0
30	66.8±2.7	93.6±1.3	47.3±1.4	30.3±2.8	7.3±1.2	9.2±0.2	2.2±0.2	84.6±2.8	1.8±0.2	31.7±1.1
60	67.5±1.4	95.6±0.9	49.5±5.1	37.0±1.1	11.9±0.7	12.3±0.6	4.6±0.3	94.9±5.0	8.4±0.9	32.7±1.6

**Hydrophobicity (%) Xylene 30 °C**										
**Time (min)**	**23V**	**33V**	**36V**	**37V**	**64V**	**65V**	**66V**	**67V**	**68V**	**73V**
15	63.8±3.9	91.3±4.1	37.6±0.5	11.0±0.7	3.7±0.2	6.4±0.4	12.8±1.6	86.2±5.4	1.5±0.1	18.8±1.0
30	66.5±1.5	93.5±1.0	42.2±0.8	21.0±0.2	5.3±0.1	8.2±0.5	19.2±1.5	93.2±3.1	1.8±0.2	25.3±2.4
60	66.9±2.9	95.4±2.5	48.1±1.1	26.6±1.4	8.6±1.0	9.0±0.9	24.5±3.6	95.6±1.3	2.3±0.5	30.9±1.9
