# Supplementary material for: Probiotic Potentiality from Versatile Lactiplantibacillus plantarum Strains as Resource to Enhance Freshwater Fish Health
Source: Microorganisms. 2022 Feb 17;10(2):463. doi: 10.3390/microorganisms10020463 (PMC8877946; doi:10.3390/microorganisms10020463)
Supplement: Supplementary file 1 [file microorganisms-10-00463-s001.zip › Supplementary material/S4 auto-aggregation.pdf]

Table S4 Auto-aggregation (AA) at 15°C and 30°C of the *Lp. plantarum* strains (23V, 33V, 36V, 37V, 64V, 65V, 66V, 67V, 68V, 73V). The data (mean ± SD; n=3) were expressed as percentage of AA.

		Auto-aggregation %									
**Temperature (°C)**	**Time (h)**	**23V**	**33V**	**36V**	**37V**	**64V**	**65V**	**66V**	**67V**	**68V**	**73V**
15	1	0.1±0.3	0.9±0.1	1.2±0.3	0.9±0.2	1.0±0.5	1.5±0.4	2.8±1.8	0.1±0.1	0.3±0.1	17.6±1.1
	2	0.3±1.8	2.1±0.4	1.2±0.2	2.3±0.2	1.2±0.4	2.1±0.1	5.1±1.2	3.1±0.4	3.4±1.8	17.6±0.9
	5	5.0±1.3	8.2±1.0	4.3±1.0	5.5±1.1	6.6±1.9	11.0±1.1	8.4±1.8	6.6±1.0	4.4±0.9	23.5±1.0
	24	34.9±2.1	30.2±1.4	33.3±1.8	31.0±1.4	20.7±2.0	33.5±1.5	35.8±1.7	40.6±1.8	49.3±1.5	44.5±1.6
30	1	6.2±0.5	5.9±2.0	5.8±1.8	5.9±1.0	4.7±1.6	6.4±2.1	3.9±0.6	4.9±1.9	4.8±0.7	7.0±0.7
	2	9.7±1.9	10.4±1.3	11.2±1.0	9.46±1.4	7.5±1.0	8.5±1.2	6.8±1.2	9.4±1.2	7.2±2.1	11.6±1.4
	5	22.0±1.1	23.3±1.0	22.3±1.4	19.0±1.7	17.2±1.3	18.6±1.8	16.1±1.4	19.7±1.6	17.0±1.1	20.3±1.3
	24	49.4±1.8	57.0±0.9	51.9±1.2	43.7±1.6	39.2±1.1	43.4±1.7	36.4±2.0	47.0±1.5	39.8±2.2	49.1±0.5
